# Effect of PVP Coating on LiMnBO_3_ Cathodes for Li-Ion Batteries

**DOI:** 10.3390/ma13235528

**Published:** 2020-12-03

**Authors:** Bolong Hong, Xiangming He, Huihua Yi, Chenglin Hu

**Affiliations:** 1 Hubei Key Laboratory of Mine Environmental Pollution Control & Remediation, School of Materials Science and Engineering, Hubei Polytechnic University, Huangshi 435003, China; hongbolong@stu.hbpu.edu.cn (B.H.); yihuihua@hbpu.edu.cn (H.Y.); 2 Institute of Nuclear and New Energy Technology, Tsinghua University, Beijing 100084, China; hexm@tsinghua.edu.cn

**Keywords:** lithium-ion batteries, polyvinyl pyrrolidone, carbon coating, MnO

## Abstract

LiMnBO_3_ is a potential cathode for Li-ion batteries, but it suffers from a low electrochemical activity. To improve the electrochemical performance of LiMnBO_3_, the effect of polyvinyl pyrrolidone (PVP) as carbon additive was studied. Monoclinic LiMnBO_3_/C and LiMnBO_3_-MnO/C materials were obtained by a solid-state method at 500 °C. The structure, morphology and electrochemical behavior of these materials are characterized and compared. The results show that carbon additives and ball-milling dispersants affect the formation of impurities in the final products, but MnO is beneficial for the performance of LiMnBO_3_. The sample of LiMnBO_3_-MnO/C delivered a high capacity of 162.1 mAh g^−1^ because the synergistic effect of the MnO/C composite and the suppression of the PVP coating on particle growth facilitates charge transfer and lithium–ion diffusion.

## 1. Introduction

Over the last two decades, polyanionic compounds, such as phosphates, sulfates, silicates and borates, have been investigated extensively as a new class of cathodes for lithium–ion batteries due to their high safety depending on their stable three-dimensional framework [1,2,3]. Among these compounds, LiMnBO_3_ has attracted much attention owing to its high theoretical capacity and low cost [1,4]. LiMnBO_3_ exists in two polymorphs, the monoclinic phase and the hexagonal phase. Monoclinic LiMnBO_3_ (m-LiMnBO_3_) occurs at low temperatures, which transforms into hexagonal LiMnBO_3_ (h-LiMnBO_3_) at high calcination temperatures [5,6,7,8]. The two polymorphs have the same theoretical capacity of 222 mAh g^−1^, while h-LiMnBO_3_ has a higher redox voltage and m-LiMnBO_3_ has better electrochemical activity [5]. More studies focus on the improvement of m-LiMnBO_3_ [9,10,11,12].

Monoclinic LiMnBO_3_ polymorph was first synthesized by Bondareva in 1978 [13]. It was discovered to have a reversible capacity in 2001, but only 2% of its theoretical capacity could be delivered, which is attributed to large polarization [14]. To improve the performance of LiMnBO_3_, several approaches have been proposed, including carbon coating [15,16,17,18,19], particle size reduction [20,21,22,23], and cation doping or substitution [9,24,25,26]. Although the performance of LiMnBO_3_ can be enhanced by doping and fine particles, it needs to be coated with carbon at the same time [15,16,17,18,19]. Carbon coating has played a key role in improving electrode materials. It could not only enhance the electronic and ionic conductivity of materials, but could also inhibit crystal growth [15,16,17,18,19].

Carbon coating is a surface modification. The carbon content directly affects the performance of LiMnBO_3_. To obtain high-rate performance, more carbon is required. However, a large amount of carbon will cause the energy density of electrode materials to decrease. To form highly conductive substances in situ on the surface of the materials is an alternative approach [27]. In addition, the effect of carbon coating depends on carbon sources and synthesis methods. Polyvinyl pyrrolidone (PVP) has outstanding wetting properties and easily forms films, making it useful as a coating additive. The pyrolysis of PVP under inert atmosphere can generate a uniform carbon coating layer on the surface of active materials, thereby improving the electrochemical performance of the electrode materials [28,29]. In this work, LiMnBO_3_/C and LiMnBO_3_-MnO/C composites were synthesized by a simple solid-state method using PVP as the additive. The effect of PVP on the structure, morphology and electrochemical behavior of LiMnBO_3_ was studied.

## 2. Materials and Methods

### 2.1. Synthesis of Materials

Carbon-coated LiMnBO_3_ materials were prepared by a solid-state reaction. Stoichiometric amounts of Li_2_CO_3_ (AR, Sinopharm Chemical Reagent Co. Ltd, Shanghai, China), MnCO_3_ (AR, Aladdin, Shanghai, China), and H_3_BO_3_ (AR, Aladdin, Shanghai, China) were added into a stainless steel container, in which 20 wt.% of PVP (K30, GR, Sinopharm Chemical Reagent Co. Ltd, Shanghai, China) from the total weight of the raw materials was mixed with 50 mL alcohol. The PVP-containing precursor was ground by ball-milling at 400 rpm for 7 h, and then was dried in an oven at 80 °C. Subsequently, the mixture was heated at 500 °C for 10 h under an argon atmosphere. After cooling in a furnace, the final black powders (marked as LMB-PVP) were obtained. The other samples in which ethanol was replaced by water or acetone, and PVP was replaced by sucrose, starch or oxalic acid, were prepared by the same route.

### 2.2. Characterization

X-ray powder diffraction (XRD, Ultima IV, Rigaku, Tokyo, Japan) with Cu Kα radiation was used to identify the phases of the samples. The morphology was observed with field-emission scanning electron microscope (FESEM, JSM-7610F Plus, JEOL, Tokyo, Japan) equipped with energy-dispersive X-ray spectroscopy (EDS, Oxford INCA, High Wycombe, UK). The synthesis temperature and carbon contents were estimated by the TGA/DSC1 thermal analyzer of Mettler Toledo (Schwerzenbach, Switzerland).

### 2.3. Electrochemical Measurements

Electrochemical measurements were performed with 2025 coin-type cells. The cells were assembled using Li metal foil as the counter electrode with a separator (Celgard 2400, Celgard, LLC., Charlotte, NC, USA) and electrolyte containing 1 M LiPF_6_ in a solvent mixture of ethylene carbonate and diethyl carbonate (1:1 by volume). The working electrodes were made by mixing active material, conductive carbon black (Super P, Timcal, Brussels, Belgium) and polyvinylidene fluoride binder (HSV900, Arkema, Pierre-Bénite, France) in a weight ratio of 8:1:1. These cells were galvanostatically charged to 4.5 V at a rate of 0.05 C (1 C = 220 mA g^−1^) and then further charged using the constant voltage mode with a cutoff current density of 0.01 C. Next the cells were discharged to 2.0 V or 1.5 V with the same rate of 0.05 C. Electrochemical impedance spectroscopy (EIS) was measured in a frequency range of 0.1–100 kHz with an alternating current signal of 5 mV. All tests were carried out at room temperature.

## 3. Results and Discussion

**Figure 1:**
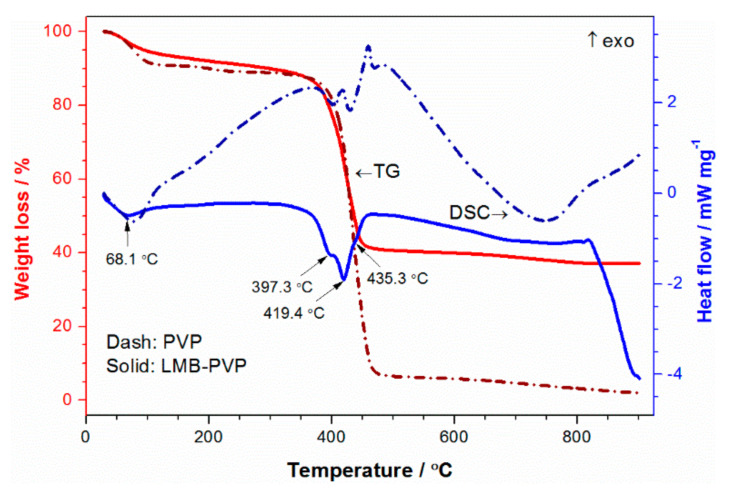
TG/DSC curves of LMB-PVP precursor and PVP measured in N_2_.

In order to obtain highly active LiMnBO_3_, the reaction temperature was evaluated by thermo-gravimetric analysis (TGA) and differential scanning calorimetry (DSC). LMB-PVP precursor was heated from 25 °C to 900 °C at a rate of 5 °C min^−1^ under a nitrogen atmosphere. Two weight loss stages and four endothermic peaks are observed from the TG/DSC curves in Figure 1. The first weight loss between 25 °C and 200 °C is attributed to the release of physically adsorbed water, corresponding to an obvious endothermic peak at 68.1 °C. Sharp weight loss appears at 300~500 °C, corresponding to three endothermic peaks at 397.3, 419.4 and 435.3 °C. The peaks at 397.3 and 435.3 °C are related to the melting and decomposition of PVP, so the crystallization temperature of LiMnBO_3_ is about 420 °C. The weight loss of the LMB-PVP precursor mainly occurs below 500 °C. Thus, 500 °C was chosen as the synthesis temperature.

**Figure 2:**
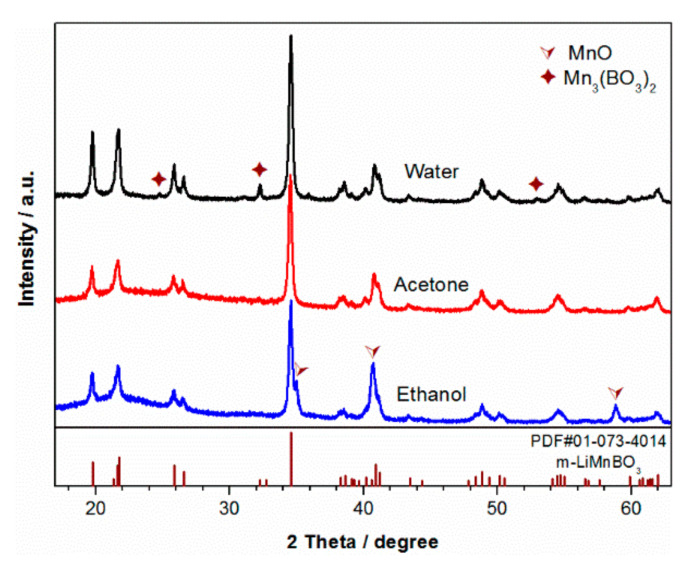
XRD patterns of LiMnBO_3_ prepared by ball-milling the raw materials with PVP in different dispersants.

The crystal phase of the samples synthesized at 500 °C was identified from the XRD experiments. The XRD patterns of the samples that were synthesized using PVP as the carbon additive in different dispersants are shown in Figure 2. All the samples have a similar pattern, which can be indexed into the monoclinic structure with the *C2/c* space group. Except for the main phase of m-LiMnBO_3_, MnO impurity phase was found in the sample with ethanol as dispersant (LMB-PVP), and Mn_3_(BO_3_)_2_ impurity phase was found in the sample with water as dispersant (LMB-W). Single phase m-LiMnBO_3_ was obtained from the sample using acetone as dispersant (LMB-A). A packet around 2θ = 18–25° is observed from sample LMB-A and sample LMB-PVP, implying the presence of an amorphous phase composition. The residual carbon of PVP pyrolyzed in an argon atmosphere was estimated by TG to be 6.86 wt.%, while no carbon diffraction peaks are seen in all samples because the carbon is in an amorphous state.

**Figure 3:**
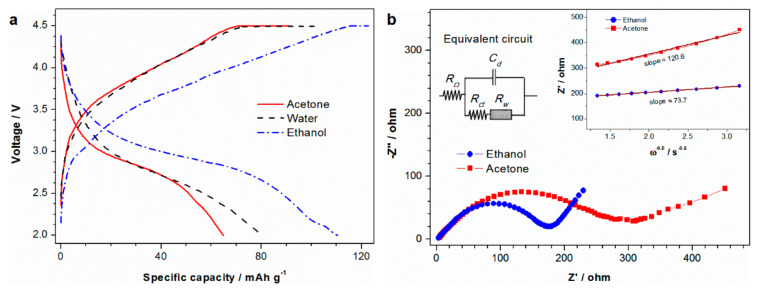
(**a**) Typical charge/discharge curves and (**b**) Nyquist plots (inset: the linear fitting of Zʹ versus ω^−1/2^ relationship) of LiMnBO_3_ prepared by ball-milling the raw materials with PVP in different dispersants.

The electrochemical behavior of the samples was measured using 2025 coin-type cells. Figure 3a shows the typical charge and discharge curves of LiMnBO_3_ samples at 0.05 C. No noticeable charge/discharge plateau is observed as in the previous report [30], indicating that the process of lithiation and delithiation is a solid solution reaction. The discharge capacities of samples LMB-A, LMB-W and LMB-PVP are 64.8, 80.1 and 110.2 mAh g^−1^, respectively. Sample LMB-PVP exhibits a better electrochemical performance due to the formation of the LiMnBO_3_-MnO/C composite. Although MnO is a semiconductor with low conductivity, the combination of MnO and carbon makes the MnO/C composite demonstrate excellent rate performance [31,32,33]. The MnO/C composite forms a good contact on the surface of LiMnBO_3_, thereby improving the electrochemical performance.

The electrochemical impedance spectra of samples LMB-A and LMB-PVP were measured in the discharged state after 10 full charge–discharge cycles. As shown in Figure 3b, the two samples have similar Nyquist plots composed of a high-to-medium frequency semicircle and a low-frequency straight-line. An electrochemical cell can be considered an equivalent circuit composed of the ohmic resistance (*R_Ω_*), the electric double-layer capacitance (*C_d_*), the charge transfer resistance (*R_ct_*) and the Warburg impedance (*Z_w_*) [34]. At high frequencies, *Z_w_* becomes unimportant and the electrode process is controlled by kinetics. As such, the semicircle in the high frequency region is related to the charge transfer process. Sample LMB-PVP exhibits smaller charge transfer resistances. The inclined line in the low-frequency region of the plots is related to lithium–ion diffusion, where the electrode process is dominated by Warburg impedance. The lithium–ion diffusion coefficient (*D*) can be roughly calculated according to the formula of $D=0.5{\left[RT/\left(AF^{2}C\sigma \right)\right]}^{2}$, where *R* is the gas constant, *T* is the absolute temperature, *A* is the cathode electrode area, *F* is the Faraday constant, *C* is the concentration of lithium-ion in the electrode and *σ* is the Warburg factor. The value of *σ* can be obtained from the linear fitting of the real part (Zʹ) versus ω^−1/2^ in the low frequency region [35,36]. The diffusion coefficients of samples LMB-A and LMB-PVP at room temperature are 2.50 × 10^−15^ cm^2^ s^−1^ and 6.69 × 10^−15^ cm^2^ s^−1^, respectively. The above results demonstrate that the introduction of MnO can facilitate charge transfer and lithium–ion diffusion.

**Figure 4:**
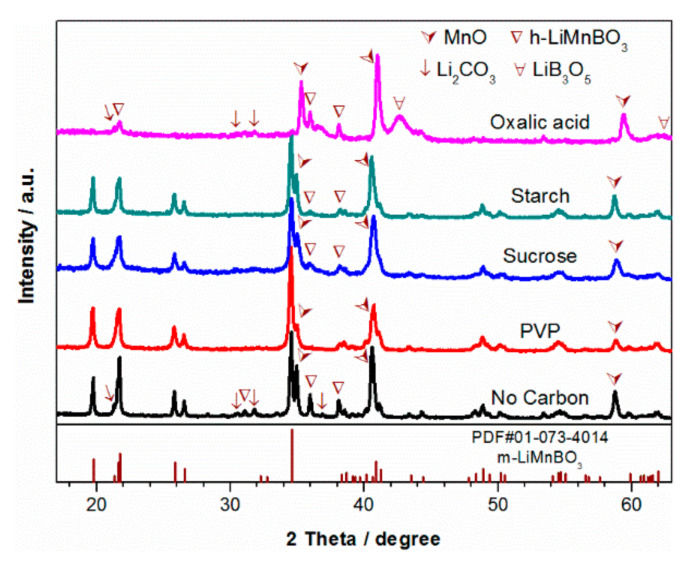
XRD patterns of LiMnBO_3_ prepared by ball-milling the raw materials with various carbon additives and without carbon in ethanol.

Different carbon-coated samples and a no-carbon sample were also synthesized by the same process. Figure 4 shows the XRD patterns of these samples. Strong diffraction peaks of m-LiMnBO_3_ are observed for all samples, except for the one using oxalic acid as the carbon additive (LMB-OA). No obvious diffraction peaks of h-LiMnBO_3_ are found in sample LMB-PVP, but they appear in the other four samples. MnO impurities are seen in all samples, implying that there are lithium and boron compounds in these samples due to stoichiometric raw materials. The sample without carbon coating (LMB-NoC) shows that a small amount of Li_2_CO_3_ did not react. Because of the strong reducibility of oxalic acid, MnO becomes the main phase of sample LMB-OA, leading to only a bit of LiMnBO_3_ in the final product. The carbon-coated sample with starch (LMB-ST) and the one with sucrose (LMB-SU) have very similar diffraction profiles. Although no other impurity peaks except MnO are detected from the XRD pattern of sample LMB-PVP, there may be some amorphous lithium and boron compounds owing to stoichiometric amounts of the raw materials.

**Figure 5:**
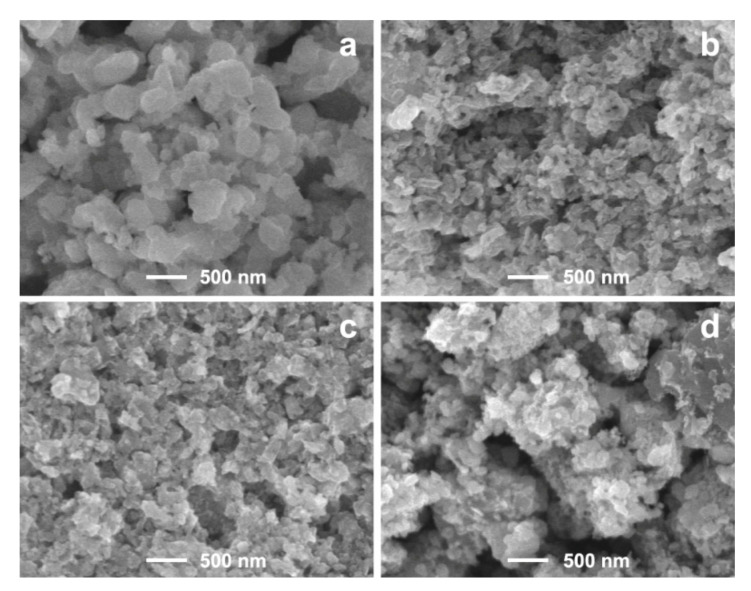
SEM images of LiMnBO_3_ prepared by ball-milling the raw materials with (**a**) no carbon, (**b**) PVP, (**c**) sucrose and (**d**) starch in ethanol.

The surface structure of LiMnBO_3_ samples was investigated by SEM. Figure 5 shows the SEM images of LiMnBO_3_ without carbon and with different carbon coatings. The sample without carbon presents an irregular spherical shape with the size ranging from 100 to 400 nm, and carbon-coated samples show smaller particle sizes of around 100 nm. Obviously, the addition of carbon suppressed particle growth of LiMnBO_3_. It is advantageous to improve the electrochemical performance of electrode materials. As can be seen in Figure 5c,d, the sample LMB-PVP has the smallest particle size, consisting of particles with a size of ~60 nm and uniform distribution. The starch sample has a particle size about 50–150 nm, which shows obvious agglomeration.

**Figure 6:**
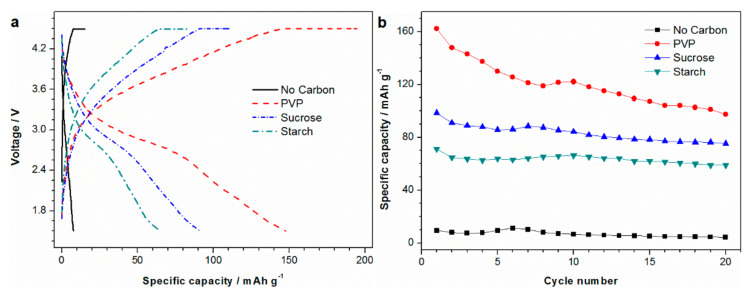
(**a**) Charge/discharge curves and (**b**) cycle performance of LiMnBO_3_ prepared by ball-milling the raw materials with various carbon additives in ethanol.

Figure 6a shows the second charge and discharge curves of LiMnBO_3_ samples at 0.05 C rate. The sample without carbon coating delivered a capacity less than 10 mAh g^−1^ with a large polarization. The addition of carbon reduced polarization and enhanced the capacity significantly. The discharge capacities of LMB-ST, LMB-SU and LMB-PVP are 64.8, 91 and 147.7 mAh g^−1^, respectively. The improved electrochemical behavior of carbon-coated samples is attributed to the increase in conductivity and the decrease in particle sizes by the added carbon. Sample LMB-PVP exhibits the best capacity among these samples due to it having less impurities and smaller particle sizes, which is consistent with the results of XRD and SEM.

The cyclic performance of LiMnBO_3_ samples at 0.05 C is shown in Figure 6b. It can be seen clearly that the sample without carbon coating reveals a low capacity, and this may be related to air exposure [7]. The initial discharge capacities of LMB-ST, LMB-SU and LMB-PVP are 71.2, 98.5 and 162.1 mAh g^−1^, respectively. Compared with the other carbon-coated materials, sample LMB-PVP with low carbon content exhibits a high discharge capacity, as shown in Table 1. The presence of MnO/C composite enhances the capacity of the LiMnBO_3_ material, and it will not significantly reduce the energy density of LiMnBO_3_ due to the high density of MnO (5.37 g cm^−3^) [37]. After 20 cycles, the capacity retention is only 60% for sample PVP. The irreversible cycling behavior is likely due to considerable Li extraction causing an unstable structure in LiMnBO_3_ [11]. Although the cycle performance of LiMnBO_3_ needs further improvement, the sample LMB-PVP still has the best electrochemical performance among all the samples.

## 4. Conclusions

Carbon-coated LiMnBO_3_ materials were synthesized by a simple solid-state method using different carbon additives in various dispersants. A LiMnBO_3_-MnO/C composite was obtained with PVP as the carbon source, which exhibits good electrochemical performance because the synergistic effect of the MnO/C composite and the suppression of the PVP coating on the particle growth facilitates charge transfer and lithium-ion diffusion. The formation of an MnO/C composite by the PVP coating can be used as an alternative surface modification approach to avoid a significant decrease in the energy density of LiMnBO_3_ coated by only carbon. The future research will focus on improving the cycle performance of LiMnBO_3_ by controlling the content of MnO.

## Figures and Tables

**Table 1:** Comparison of the discharge capacities of LiMnBO_3_/C at 0.05 C.

Sample	Carbon Resource	Residual Carbon (wt.%)	Discharge Capacity (mAh g^−1^)
This work	PVP	6.9	162
Ref. [7]	Ketjen black	10	~70
Ref. [12]	sucrose	<10	102
Ref. [16]	Ketjen black	22.6	150
Ref. [30]	Ketjen black	~10	~170
